# Case report: A novel *de novo* loss of function variant in the DNA-binding domain of TBX2 causes severe osteochondrodysplasia

**DOI:** 10.3389/fgene.2022.1117500

**Published:** 2023-01-17

**Authors:** Misbahuddin M. Rafeeq, Hussam Aly Sayed Murad, Najumuddin, Samee Ullah, Zaheer Ahmed, Qamre Alam, Muhammad Bilal, Alaa Hamed Habib, Ziaullah M. Sain, Muhammad Jawad Khan, Muhammad Umair

**Affiliations:** ^1^ Department of Pharmacology, Faculty of Medicine, Rabigh, King Abdulaziz University, Jeddah, Saudi Arabia; ^2^ National Center for Bioinformatics (NCB), Quaid-i-Azam University, Islamabad, Pakistan; ^3^ National Center for Bioinformatics (NCB), Islamabad, Pakistan; ^4^ Department of Biosciences, COMSATS University, Islamabad, Pakistan; ^5^ Molecular Genomics and Precision Medicine, ExpressMed Laboratories, Zinj, Bahrain; ^6^ Department of Biochemistry, Quaid-i-Azam University, Islamabad, Pakistan; ^7^ Department of Physiology, Faculty of Medicine, King Abdulaziz University, Jeddah, Saudi Arabia; ^8^ Department of Microbiology, Faculty of Medicine, Rabigh, King Abdulaziz University, Jeddah, Saudi Arabia; ^9^ Department of Life Sciences, School of Science, University of Management and Technology (UMT), Lahore, Pakistan; ^10^ Medical Genomics Research Department, King Abdullah International Medical Research Center (KAIMRC), King Saud Bin Abdulaziz University for Health Sciences, Ministry of National Guard Health Affairs (MNGH), Riyadh, Saudi Arabia

**Keywords:** TBX2, novel variant, nonsense mutation, chondrodysplasia, WES

## Abstract

**Background:** T-box family members are transcription factors characterized by highly conserved residues corresponding to the DNA-binding domain known as the T-box. TBX2 has been implicated in several developmental processes, such as coordinating cell fate, patterning, and morphogenesis of a wide range of tissues and organs, including lungs, limbs, heart, kidneys, craniofacial structures, and mammary glands.

**Methods:** In the present study, we have clinically and genetically characterized a proband showing a severe form of chondrodysplasia with developmental delay. Whole-exome sequencing (WES), Sanger sequencing, and 3D protein modeling were performed in the present investigation.

**Results:** Whole-exome sequencing revealed a novel nonsense variant (c.529A>T; p.Lys177*; NM_005994.4) in TBX2. 3D-TBX2 protein modeling revealed a substantial reduction of the mutated protein, which might lead to a loss of function (LOF) or nonsense-mediated decay (NMD).

**Conclusion:** This study has not only expanded the mutation spectrum in the gene *TBX2* but also facilitated the diagnosis and genetic counseling of related features in affected families.

## Introduction

The *TBX* gene family encodes key factors in embryonic development, organogenesis, and tissue homeostasis. The T-box protein family shares a conserved T-box domain that binds DNA in a sequence-specific manner and functions as transcriptional repressors and/or activators (Ouimette et al., 2010). The members of the T-box gene family are found in all metazoans, ranging from hydra to humans, and are notable for the crucial roles they fulfill during embryonic development (Adell et al., 2003; Showell et al., 2004).

The Tbx2 transcription factor, a member of the T-box family of proteins, has been associated with human skeletal and cardiac abnormalities (Radio et al., 2010). In mice, Tbx2 is mapped on chromosome 11 (Bollag et al., 1994), while in humans, TBX2 is located on chromosome 17q23. The T-box domain of the human TBX2 shares 90% DNA and 96% peptide sequence homology with its mouse counterpart. Both human TBX2 and mouse Tbx2, which span 3,378 bp and 3,562 bp, respectively, comprise seven exons. Similarly, the homozygous Tbx2 knockout mice exhibit atrioventricular canal defects, pericardial edema, cleft palate, polydactyly, and embryonic lethality, which indicates the crucial role of Tbx2 during development, particularly during cardiac development. In addition, experiments on the chick have shown that the Tbx family member [Tbx3] plays an essential role in posterior digit specification, acting together with Tbx2 and the inter-digital BMP signaling cascade. Most of the heterozygous variants in TBX2 have been reported in a multisystem malformation disorder with severe skeletal development defects such as vertebral anomalies and syndromic cardiovascular dysfunction (Liu et al., 2018), while only one case has been associated with osteochondrodysplasia with empty sella (Mäkitie et al., 2022). The phenotypes reported in these prior studies provide additional support for the notion that the severe skeletal defects observed in our patients are caused by disease-causing variants in the *TBX2* gene.

## Methods

The present family having a single affected individual (II-1) was recruited from a remote region of Pakistan. The studies involving human participants were reviewed and approved by UMT, Lahore, Pakistan, and COMSATS University, Islamabad, Pakistan. Written informed consent was obtained from the individual(s) and/or minor(s)’ legal guardian/next of kin for the publication of potentially identifiable images or data included in this article. Blood samples for DNA extraction were collected and quantified using standard methods.

### Molecular analysis

Genomic DNA of an affected family member (II-1) was subjected to WES. DNA was prepared according to the Agilent SureSelect target enrichment kit preparation protocols/guidelines. WES was performed commercially by the College of American Pathologists (CAP) accredited molecular diagnostic lab, and the libraries were sequenced using the Illumina platform HiSeq 2000 (Umair et al., 2021a; Ullah et al., 2022). Variants identified in the present study were verified using bi-directional Sanger sequencing using standard methods (Hayat et al., 2020). The primers for Sanger sequencing were designed using the Primer3 online tool (genomic build number: GRCh38/hg38) (*TBX2*: Gene ID: 6909; Transcript ID: NM_005994.4) (Supplementary Material S1).

### Computational analysis

Several databases, including gnomAD, dbSNP, All of Us, and Bravo, were screened to search for the selected variants. The pathogenicity of the identified variants was analyzed using online bioinformatics tools: MutationTaster, CADD-Phred, GERP++, VarSome, BayesDel addAF, DANN, Eigen, FATHMM-MKL, and LRT.

### Structure modeling and molecular dynamic simulations

In this study, the three-dimensional (3D) structure of TBX2^WT^ (T-box transcription factor 2) was obtained using I-TASSER and homology modeling with MODELLER (Ahmad et al., 2018; Khan et al., 2020). As there was no known X-ray crystallography-based 3D structure available, the protein sequence (NCBI Accession: NP_005985.3) was obtained from the National Center for Biotechnology Information (NCBI) database (https://www.ncbi.nlm.nih.gov/) and used as the input for I-TASSER. The resulting 3D structure was then used as a template for predicting the structure of TBX2^LYS177TER^.

Both TBX2^WT^ and TBX2^LYS177TER^ were subsequently subjected to molecular dynamic (MD) simulations using Desmond version 2.3. The simulation conditions included setting the box boundary coordinates to 10 Å, with a volume of 1200688 Å³ for TBX2^WT^ and 617233 Å³ for TBX2^LYS177TER^. The salt concentration was set to .1 M for both systems, and the systems were neutralized by adding 14 Cl ions to TBX2^WT^ and 1 Cl ion to TBX2^LYS177TER^. The resulting systems contained 89176 atoms (TBX2^WT^) and 5778 atoms (TBX2^LYS177TER^). The simulation time was set to 100 ns, and the ensemble class was set to NPT, with a temperature of 300 K and a pressure of 1 atm. The default “relax model before simulation” protocol was used, and trajectories were generated for the duration of the simulation.

## Results

### Clinical description

**FIGURE 1:**
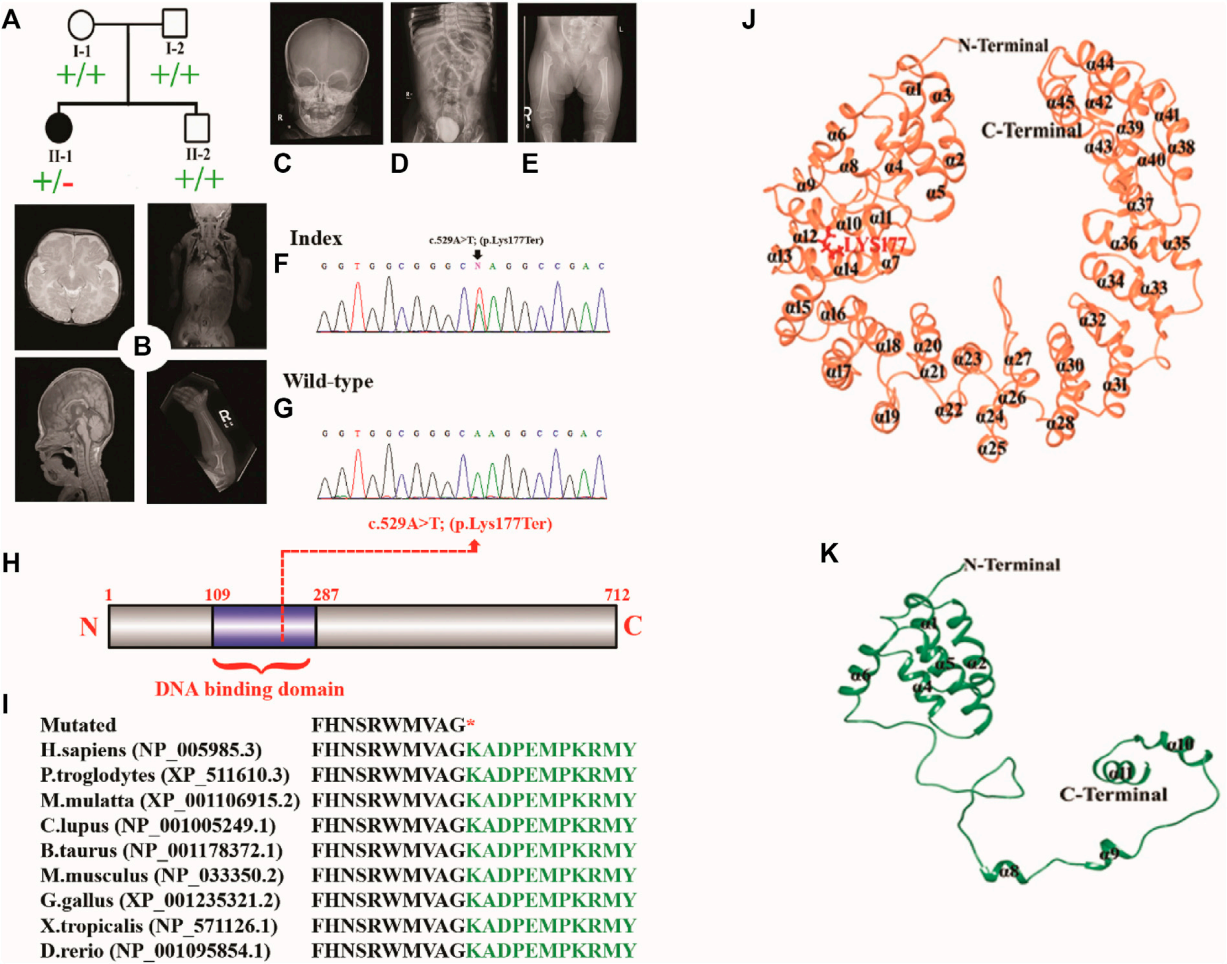
**(A)** Pedigree of the investigated family. **(B–E)** MRI and radiographs of the proband showing platybasia at the skull base, delayed myelination for the patient’s age, and severe skeletal deformities. **(F,G)** Sanger sequencing electrograms of the proband and wild-type. **(H)** Schematic diagram of the TBX2 protein showing the position of the identified variant. **(I)** Partial amino acid sequence of the TBX2 protein showing conservation of Lys177 across different species. **(J, K)** Mutational and structural analyses of TBX2. Structural differences between **(J)** TBX2^WT^ (coral) and **(K)** TBX2^LYS177TER^ (sea green). All the secondary structures, N-terminal, and C-terminal are labeled in black color. Lys177 is shown and labeled in red color.

The proband was a five-year-old female patient (deceased). The proband (II-1) had the clinical features such as chondrodysplasia, short stature, and global developmental delay (Figure 1A).

### Brain CT-MRI

The midline structures, including the pituitary gland and suprasellar regions, were normal. The foramen magnum is unremarkable. There was no evidence of basilar invagination. The base of the skull angle measures 179°, suggestive of platybasia. The gray/white matter differentiation was preserved. There was delayed myelination for the patient’s age. No definite cortical deformity was identified. Posterior fossa structures were normal with no evidence of Chiari 1 malformation. Prominent and extra-axial CSF spaces with prominent ventricular consistency were observed at infancy. There was no hydrocephalus, hemorrhage, or space-occupying lesion. Posterior fossa structures were normal with no Chiari I change. The visualized parts of the orbit, paranasal sinuses, and labyrinthine structures were normal. There was occurrence of platybasia at the skull base, and delayed myelination for the patient’s age was observed (Figure 1B).

### Renal ultrasound

The right kidney measures 6.7 cm (previously 6.5 cm), and the left kidney measures 7 cm (previously 6 cm). Both kidneys demonstrated normal parenchymal echogenicity and good corticomedullary differentiation with multiple non-obstructed stones. Persistent bilateral non-obstructing renal stones were more on the right side. Grade 1 right hydronephrosis was observed, along with urinary bladder debris correlating with urinalysis laboratory values.

### Abdomen and pelvic CT

The enteric feeding tube was placed in the proper position within the stomach. A double stoma was observed in the left upper quadrant. The afferent and efferent loops were observed to be collapsed. The contrast was observed in the efferent loop opacifying the sigmoid colon. The significant proximal jejunum, duodenum, and gastric dilatation were related to high-grade mechanical bowel obstruction. The distal bowel loops were collapsed. The maximal diameter of the small bowel that courses behind the mesentery into the left abdomen was 3.1 cm. However, it was difficult to assess the proximal continuity of the bowel loop. This could be related to an internal hernia. The pelvis had minimal free fluid and several non-obstructive right renal stones. The largest stone recorded was of size .8 cm. The visualized chest parts showed bilateral subsegmental atelectasis/consolidation in the lower lobes. The liver, spleen, pancreas, and adrenals were unremarkable (Figures 1B–E).

### X-ray findings

The affected individual manifested widened irregular metaphysis with more destructive changes at the proximal femoral ends. In addition, there was a bilateral superolateral displacement of the proximal femur and stippled calcification along the knee joints. Levoscoliosis in the thoracolumbar spine and symmetrical limb shortening were observed. All these skeletal changes favor the chondrodysplasia punctata type of skeletal dysplasia (Figures 1B–E).

### Genetic findings

DNA of the affected member (II-1) was subjected to WES using standard methods (Ahmad et al., 2018; Hayat et al., 2020; Khan et al., 2020; Umair et al., 2021b). The variant filtration criteria were based on the gnomAD frequency of ≤.0001, CADD-Phred score of ≥15, exonic and splice site (+/− 12 bp) variants, and Kaviar allele count of ≤10. A *de novo* nonsense variation in the *TBX2* gene (c.529A>T; p.Lys177*; NM_005994.4) was detected which was validated by Sanger sequencing (Figures 1F, G). The variant had a CADD-Phred score of 39 and a GERP++ score of 4.81. Combined Annotation-Dependent Depletion (CADD) and other associated tools are widely used to measure the disease-causing nature of variants that can effectively prioritize causal variants in genetic analyses. These tools are integrative annotation built from several genomic features and can score human single-nucleotide variants and short insertion and deletions effectively. The Lys amino acid at position 177 was highly conserved across different species (Figures 1H, I). According to ACMG, the identified variant was classified as likely pathogenic (Class 2).

### Structural analysis of TBX2^WT^ and TBX2^LYS177TER^

Structural analysis of TBX2^WT^ and TBX2^LYS177TER^ revealed several differences between the two proteins. Specifically, in TBX2^LYS177TER^, the α2 and α3 regions were fused together to form a single α2 region, while the α7 region was converted into a loop. Additionally, the loop between α7 and α8 was converted into a helix (designated α8 in TBX2^LYS177TER^), and the α11 region was extended from Lys166 to Ala175 (Figures 1J, K). These structural changes were unique to TBX2^LYS177TER^ and were not present in TBX2^WT^.

### Conformational dynamics

**FIGURE 2:**
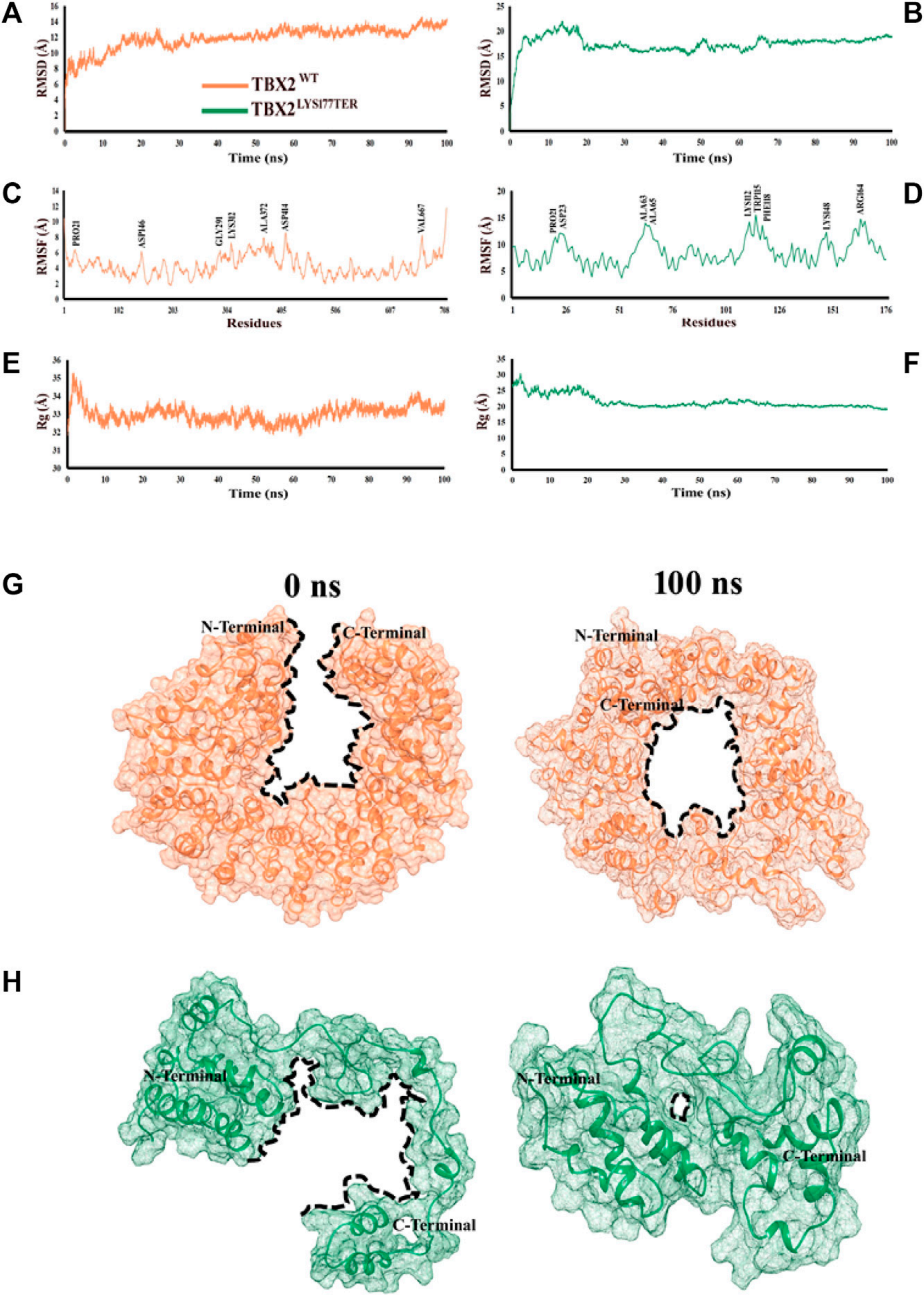
Time-dependent RMSD, RMSF, and Rg analyses of TBX2WT and TBX2LYS177TER over the 100 ns simulation time. **(A, B)** RMSD plot over the function of time. **(C, D)** RMSF plot over the function of time. **(E, F)** Radius of gyration plot over the function of time. TBX2^WT^ is depicted in coral, while TBX2^LYS177TER^ is depicted in sea green color. **(G, H)** Conformational changes in TBX2^WT^ and TBX2^LYS177TER^ over the 100 ns simulation time. **(G)** Conformational changes in TBX2^WT^ at 0 and 100 ns. **(H)** Conformational changes in TBX2^LYS177TER^ at 0 and 100 ns. TBX2^WT^ is depicted in coral, and TBX2^LYS177TER^ is depicted in sea green color. The black dotted lines signify the cavities. N- and C-terminals are depicted in black color.

The root mean square deviation (RMSD) is a measure of the stability of a protein structure over time. In this study, the observed RMSD values of 8–12 Å for TBX2^WT^ and 10–15 Å for TBX2^LYS177TER^ suggest that both systems displayed relatively stable behaviors during the simulation. However, the higher RMSD values for TBX2^LYS177TER^ may indicate that this protein is less stable than TBX2^WT^. This difference in stability may be due to structural differences between the two proteins, such as the fusion of the α2 and α3 regions and the conversion of the α7 region into a loop in TBX2^LYS177TER^, which may introduce additional conformational flexibility into the protein. The analysis of fluctuating amino acids also indicates that TBX2^LYS177TER^ is less stable than TBX2^WT^, as TBX2^LYS177TER^ exhibits increased fluctuation in certain amino acids, including Pro21, Asp23, Ala63, Ala65, Lys112, Trp115, Phe118, Lys148, and Arg164. These structural changes, such as the extension of the α11 region and the formation of a new helical region (α8) in TBX2^LYS177TER^, may also contribute to the increased fluctuation in certain amino acids. Additionally, the radius of gyration, which is a measure of the compactness of a protein structure, indicates that TBX2^WT^ has a more compact structure than TBX2^LYS177TER^. The observed radius of gyration values of 33–35 Å for TBX2^WT^ and 25–30 Å for TBX2^LYS177TER^ suggest that TBX2^WT^ has a more tightly packed structure. This difference in the packing density may also contribute to the overall stability of the proteins, as a more compact structure may be less prone to conformational changes. In summary, the observed differences in RMSD values, fluctuation in amino acids, and radius of gyration values suggest that TBX2^LYS177TER^ is less stable than TBX2^WT^. At the 0 ns time scale, both TBX2^WT^ and its mutant form are in open conformations. However, upon reaching the 100 ns time scale, both proteins exhibit a shift toward close conformations. This change is likely due to the close contact between the N- and C-terminals over the course of the simulation. As a result of this interaction, TBX2^WT^ assumes a circular shape, with the inner side forming a circular cavity (as depicted in Figures 2G, H). It is worth noting that the shift from open to close conformations may have different functional consequences for the wild-type and mutant forms of TBX2. Further investigation is necessary to fully understand the impact of these conformational changes on the function of these proteins.

## Discussion

Recently, Mäkitie et al. (2022) associated the heterozygous variant (c.899C>T; p.Thr300Met) in the *TBX2* gene with chondrodysplasia. In the current study, using WES, we identified a novel *de novo* nonsense variant (c.529A>T; p.Lys177*; NM_005994.4) in the exon 2 of the *TBX2*. This variant was classified as likely pathogenic (Class 2) by the American College of Medical Genetics and Genomics (ACMG), and multiple online prediction tools indicated that this variant is likely to be disease causing.

In the present study, the proband showed overlapping clinical features of chondrodysplasia. Unlike the clinical features of the patient reported by Mäkitie et al. (2022), the proband presented here showed spinal deformities, including short limbs, metaphyseal and epiphyseal dysplasia, and bilateral developmental dislocation of the hip (DDH), which expands the phenotypic spectrum of *TBX2*-associated variants. Furthermore, developmental delay was observed which was not reported previously. The diversity of the phenotypic presentation might be due to the position and nature of the mutation. Our patient had a nonsense variant that might lead to mRNA nonsense-mediated decay; however, the patient reported by Mäkitie et al. (2022) revealed a missense variant.

The variant identified in the present study (c.529A>T; p.Lys177*) resides in the T-box DNA-binding domain of *TBX2*, which is located at amino acids 106–289 [mice and human]. The T-box DNA-binding domains exist in many organisms, including nematodes, frogs, chicks, mice, and humans, associated with growth control and many aspects of organogenesis (Minguillon and Logan, 2003; Naiche et al., 2005; Abrahams et al., 2010). Therefore, it is highly likely that it removed approximately one-fourth of the protein and abolished its function or it might have resulted in complete degradation of the transcript through nonsense mRNA-mediated decay (NMD).

Variants in *TBX2* of both single nucleotides and more significant copy number variations (CNVs) have been associated with skeletal disorders, development delay, cardiac defects, endocrine disorders, craniofacial dysmorphism, and immune deficiencies (Radio et al., 2010; Nimmakayalu et al., 2011; Liu et al., 2018; Mäkitie et al., 2022). The most common features associated with skeletal deformities in TBX2 are spinal deformities such as kyphosis, scoliosis, and Klippel−Feil anomaly. In addition, limb and rib anomalies were also observed (Radio et al., 2010; Nimmakayalu et al., 2011; Liu et al., 2018).

Tbx2 and Tbx3 form a second pair of structurally related T-box genes with an essential function in limb patterning. Tbx2 and Tbx3 are expressed in the anterior and posterior edges of limb buds with distinct domains in relation to the posterior digit identity. Tbx2 expression in the anterior and posterior limb mesenchyme depends on the presence of the non-AER DV border ectoderm. In contrast, the AER–FGFs inhibit Tbx2 expression, explaining the regulation behind its striped pattern (Lecanda et al., 2000; Chen et al., 2004; Nissim et al., 2007).

Genetic disorders such as chondrodysplasia are very severe and usually result in death. In such a scenario, proper genetic counseling and the introduction of new genes/variants to newborn screening programs and parenteral diagnosis can play a major role in reducing the burden of such severe disorders (Alfadhel et al., 2019). This can be accomplished by prenatal genetic testing for monogenetic disorders (PGT-M). PGT and *in vitro* fertilization are options for parents wishing to have future pregnancies (Alyafee et al., 2021a; Alyafee et al., 2021b; Alyafee et al., 2022). Although WES has revolutionized the clinical molecular diagnosis of many unsolved genetic disorders, still more functional research is required on such novel disease gene associations (Umair et al., 2018). There is no specific management for such severe skeletal disorders; however, affected individuals are treated with supportive treatment (Umair et al., 2019).

In conclusion, we report a proband with chondrodysplasia and GDD due to nonsense variants in *TBX2*. Our findings support the recent observation that the heterozygous variant in *TBX2* might be associated with chondrodysplasia in humans. Further studies are required to clarify the variable phenotypes associated with *TBX2* variants. However, further investigation is necessary to fully understand the diverse range of phenotypes associated with TBX2 variations.

## Data Availability

The datasets for this article are not publicly available due to concerns regarding participant/patient anonymity. Requests to access the datasets should be directed to the corresponding author.
