# Supplementary material for: Case report: A novel de novo loss of function variant in the DNA-binding domain of TBX2 causes severe osteochondrodysplasia
Source: Front Genet. 2023 Jan 17;13:1117500. doi: 10.3389/fgene.2022.1117500 (PMC9888409; doi:10.3389/fgene.2022.1117500)
Supplement: Supplementary file 1 [file DataSheet1.PDF]

TBX2 Sequence and position of primers

TCCCGCCACCTGGGATCCGCTCCCGGACCTCGGCCGCCCGAGCCTCCCGACTCGCCCGCC
CACCGGCCTCGCTTTCCAGTGCCTGCTGTCTCTTTCCGGGGCGCAGGGACCCCCAGGCGG
CGCCACGACCCGGAGCTGGGTCGGAGGCCAGGCTGGGGCTCGGGCCAGAGCGGCCGGGCC
TCTGGGCGCGGGAGAAGGAGGGCGCCCCCTCTCCGCTCGGGGTCGTGTCAATGCTTTGCA
CTTGGGGCCGGCGTGCGGCTGCGGGTCCTTCCCCAAGGCCCCGGGACCCGGGCTCCCCTC
GCCTCAGGCCCTTTCGGCGGGTCAGGTCGGCCCTCCGCGCTCTCCAGTCCGGCGCAGGCA
GCCGCAGGCGCGGGCGGGCGGTGGGGGCCGGGGCAGGAGGAGGGGTCTCGGGGCCCGGCG
GCCCGTCATTGGTTAATATTTTATTCTGTTGACATGTTTTCTTACTGCTGAGGCTTCCGA
CACCTTCTCCCAGGCCCCCCCTCCCGGCCGGAGCTTGGCCTGAGCTGTCAAAACCCCGCC
CCCGGAGACCCACAATTGGTCCAAAAAGCGTAAAATCAGCAATCAAGGGGGGCCTGGCTC
GTTAGCGCAGGGGATCCGAGCTGGGCAGGACATGTGAGATAGTCACAGTTTTCCAGAGAT
CACGACAAGATCTAACCAGTCGCGCGTGGTCCCCGGCGCCGGAGCGGGCCAGCTCAGCCC
GGCCCAGCCCGGCCCCGCGCAGAGCCCCCGCCGCCCCCGCGCACAGAGCCGGGTGCCCCT
TGCGGTGCGCCGGACGGGAAGCCCCGAGGAGCAGCTGCTGCGCCCGCCACCCGGGTCGTC
CGTCCACCGCGCGCGCCGCCGCCCGGGCCGGGGGTCCGAGCCGCGCGCCCCCGGCCCCGG
CCCCGGCCCCCGGGCGCCTGGGCCGGATGTCCCGATGAGAGAGCCGGCGCTGGCGGCCAG
CGCCATGGCTTACCACCCGTTCCACGCGCCACGGCCCGCCGACTTCCCCATGTCCGCCTT
TCTGGCGGCGGCGCAGCCCTCCTTCTTCCCGGCACTCGCGCTGCCGCCCGGCGCGCTGGC
CAAGCCGCTGCCCGACCCGGGCCTGGCGGGGGCGGCGGCCGCGGCGGCGGCGGCGGCAGC
AGCGGCCGAGGCGGGGCTGCACGTCTCGGCACTGGGCCCGCACCCGCCCGCCGCGCATCT
GCGCTCCCTCAAGAGCCTGGAGCCCGAGGACGAGGTGGAGGACGACCCCAAGGTGACGCT
GGAGGCCAAGGAGCTGTGGGACCAGTTCCACAAGCTAGGCACGGAGATGGTCATCACCAA
GTCCGGGAGGTAGGGCTGCCGGCCGGCTGGAAGGCGCGCGGGCGGGCGGGCGGGCTGGGG
CACGGGACTGCACGGATCAGAGCAGAGCTGGGGACTCCCGGCTCCCGGCTCCCGGCTCCA
GGTTCTGGCCCTGACGCCACGCTTCGCTCCCACGGACAACCAAGTTGACTTTTCTCGTTT
GGCACCGGAGCGATTTTTTTTTAAAACAAAAACGCTAAAATCCTCCGAATGAAATAAAAC
GAAAACATACTGCTAAAGAATAGCCCCAACCGTTCTGAGTCCCAGCGCAGAGGAGGCCCC
GCGGCTTGGCCCTGGCGGTCTCCTCCGCCCCGCGCTCTCGCTCTTCTGCGTCCGGGTCCG
TCTCCGAGCTCGGGGGAAATTCAGCCTCTCTCAGACTCTGCTCCGACCCCGAAGCCCCTA
GTGGGACCTGGGCCCAGCTAACCCCACGCTGGTTGCTCGGTTCCTGGCAGCGAGCCCCGG
GTCAGCCGAGCCCTCCGCCTCACCGGGCTGGGAGCCCTTTCTCCACCGCGGGCATCCGAG
CCCTGGACGCATCCGCCGCCCCAGGCCTTAAAGCCTGAAGAAAGCCACAGCCCCGGCCCC
TCCACCCTTCGACTCAGCCACCGAGAATCCAGGCCTCGGGTTCACCCTCTTCCCCCGAAC
TGCACGGCCAGGATGTCTTATCAGGCTCTGTAGCCCAGTTCCCAATACAAACACCCTCCA
GATTTATTTCTGGGAGCTTCCGTCCCAAGTGGGTATTTCCCCTGAACGAATTTCGGGGAG
ATTAAGGAGAAGCGAGAATATTTCTAGAGGGCCTAGACTTCTCTCCTGGGCATCAGCTTT
CATCTCAGATCAGGGAGAAAGAGGGTCCCCAGATCTGAGCACAAGGCCTAAAGGAGGCTG
TTTAGGAGAGGGTCTGACAGGCAGAAATGGGATCTCCTGGGAGCAACAACCACAGGTGGG
GTCGTCCGGGCAGTGATGAGAGGGCAGAGCAGCCGACCACAGGGGAAACAGCCAGGCGGC
AGCGGTGTGCGCAACGAGGAGGGATAAATAAAGGAGGAGTGGGGTCCTGGAACCTAGAAC
AGCCGGTTCCAATGGGATCTCCTCCCTTCCCTCCCTCCCAG**GCGGATGTTCCCCCCCTTC**
AAGGTGCGAGTCAGCGGCCTGGACAAGAAGGCCAAGTATATCCTGCTGATGGACATTGTA
GCCGCTGACGATTGCCGCTATAAGTTCCACAACTCGCGCTGGATGGTGGCGGGCAAGGCC
GACCCTGAGATGCCCAAACGCATGTACATCCACCCAGACAGCCCAGCCACGGGGGAGCAG
TGGATGGCTAAGCCTGTGGCCTTCCACAAGCTGAAGCTGACCAACAACATCTCTGACAAG
CACGGCTTCGTGAGTGTTGGGGCAGGGTGGGGACGGTGCAGGAGCTTGTTGACCCAGCAC
TGCAGCTGAGCAGGAGAGCAGGGCGGCAGGATCTCCCAGGGGGAAGCGCTGGGCAAACCC
CCAGAGTGCCCCTGCCCGGGTCACTGCCCTGTGGTCTACGTGGGCTGGGCCTGGGCCTGG
GCCCCAGCGCTCTCCTTTGCAAGCCTGGGAAATGGTGGGGTCAGTGGCTGGCAGCTCACA
ATCTCAGTTCCTCAGGCCGTGGAGGGTCCCTTGTACCGTCAATAGGGAGGCAGGTGCTGC
CAGGACTGGGTCTGCAGTGCTGGACCACGGAGGTCACCCTGCTCTTGGCCTGTAGGTGCC
GAGCTTGGAAGACCTGCCCATGACCTCAACTTGTCCATCACCCTTCTGCCAAGACCCGCG
TCCCTGGCCCTCCCCCAAGACTAGGGGATTTAAAAGTTAAACCACCCTCCCCACCCACCC
CCCCAGCCTCCCTAAGACCTTTCCCTCCTCCCACAGGCAGATGCCCAACTAGGAGGAGAG
GGTTTGGGGGTTCTCTCCATTTATGCCTGAGCAAGGCCCCAGCGAACGGGGGTGGTGTTT
CATAGGGTAAAAGGAACTGCCCATTCTGAGGTAGGGCAGTTGGTGAGCCCCCGAAGGTTG
GAATCTCCTCTTTGTGGGGTTAGGGGGTTTACCAGTGCCAGAAAGGAAGAGAGTGTTAAG
AGTGGAATTGTAGGGCTCTGTTTTGTTTTATTTTGTTTGGGTTTCTTTTGTGGGGAGGGG
TTGTTTTTTTGGTTGGTTTTTTTGTTTTGTTTTTTGTTTTGTTTTGTTTTTGCTCTGTCTC
ATTTCTGTTTCCAACTGGGAAATTTTTTTTAATGGCAAGAGAAAAAGAAGCTGTGAAAAG
AGAAAAGCGAGAGGAAAAGTAGAAGAAAAAGAAATAAGGGTGAGAAAAGAGCAGAGACAG

TBX2 (NM_005994.4):

c.529A>T; (p.Lys177Ter)

FI-R1:

F2-R2

GAGAAAATGGGGAAGAGGAAGAACCGATAGAGAGAGAGAGAGAGAGAGAGAGAGAGAGAG
AGAGAGAGAAAGTGGAGAGGAAGAGGTCAGGTACCCAAAGAATAGAAAAGCTCGGGCCGG
GGCTGGTGGCTGCCGGCTGACCCCCACCCTCCCCGCAG**ACCATCCTAAACTCCATGCACA**
AGTACCAGCCGCGCTTCCACATAGTGCGAGCCAACGACATCCTGAAGCTGCCTTACAGCA
CCTTCCGCACCTACGTGTTCCCGGAGACCGACTTCATCGCCGTCACTGCCTACCAGAATG
ACAAGGTGCGCGCGGCGGGCGGTGGGCTAAGCCCCTGCACTGACGCCCCTCAACACGTGC
AGGCCCAACCGTCCGTTCATCGCCCCTCGAAGCCCCCTGCACGGGATCCGCGCTCTTGCG
GCCCGCCCCCGAGCACCGAGCCTCGCATCCATACGCGCAGCACTCACGGAAGTCCTCAAG
GCGCCCTCTCAATCCCACCGCGCGCACACACAGGCTCCCCTGGGGCGAGCGAACAGCTGG
GCCGTCGTTCTGATGGAGATGTTTACTTAACAATTAGCTGAGACTCCGGGCCCGCGCTGA
CATTTTTACATTTTATTCGTTTATTTCTTGGCTCAGGAATGAGAATGTAAGTGAAGAGAG
GGCGCCCGAGGCAATCTGCCCAGGCTCTACCGATGCTCAGAACCCGGGGCCCAGTTTTCA
CTCTCTCGGGGGGAGCCAGAGGGTGGGACAGGTGACACAGCCCCCCAAAGCACTCCTGGC
TACTGCTCTGGGTCAGTCTAGGCCCCAGACCCCTTGGGAGGCGCTTAGAGAATTACCTTC
CAATCAAAGGGCTTTTTCTGCTTGCGGCTTCTCCACCCTTGTTCTAGTGGGGCAGAGCAT
CACCCCTCTAGGCCTGTGCCCTTGTGCGCCACTGTCGAGTGAAAGAGAGGGTACGAGTGT
CCGTGCCGGTCGTTGTGGCTTTGTGAACCAAGGTGTACGCTGGGCTGTGCGTGCCCCGTT
TGGGGTGTGTTGGATACCTGTGGAGGCATGTGAATTCGTTTGGTTCCGTAGGCGTTTGTT
ATGTCTGTGCACACTTGCGTGTGTGCGAGCCCGTGAGTGTGATTTGGCTTTGTGTGTACC
TGCAGCCTTGCGTGTGAACTGCAGTCTCTGTTCGCGCCTGCTCGGGTGCGGAGTCGTTTA
ACTTGCGCAAGCCCGCGGGCGGGTGTGCATGCTTGGGCTGGTTCCGACACTGTGGCACTG
CTTTAGGCCTGTGTGGGGCTGTCTAGGCCTGTCTAGGGATAATTCCTCGAATGAAGCTTT
TGGAGGACAGACCCCGTCGCTTTCCGCGAGGCCCTCCCACCGGCCTCAGCTCGGGGCGTC
CCATCCCAGGCGGGTGGGTACCAAGTGGACGCTCCCCGGGTCTTCCCTCTGCGGCCAGCA
CCCGCTGACCTCGGGGTGCCACCGACCACGCTGGAAGAGCCACGGCCTGACTTAGCGCCG
CCCCCTTGGTCCCCGCAG**ATCACACAGCTGAAGATCGACAACAACCCGTTTGCCAAGGGC**
TTCCGGGACACCGGGAACGGCCGGCGGGAGAAAAGGTGAGAGGCCGAGGACAGCAGCCCT
GTGGCGGGTGCCCGCGGGAGCAGCGAGCAGGAGGGATGGAGGGATGGGCTCCCCGCTGAC
CTGGTTCATCCGCTCATCCCCAG**GAAGCAGCTGACGCTGCCGTCTCTACGCTTGTACGAG**
GAGCACTGCAAACCCGAGCGCGATGGCGCGGAGTCAGACGCCTCGTCGTGCGACCCTCCC
CCCGCGCGGGAACCACCCACCTCCCCGGGCGCAGCGCCCAGTCCGCTGCGCCTGCACCGG
GCCCGAGGTGAGGGTCGGACCGGAGGAGGGACAGGGAGGTGGCGGCGGGGGGTCCTCAGG
TCGCTGGGCTGGTCTTTTGCTGAGCCACCCGCTAACCTGAAAGGCCAGGAAGGAAACGTC
GGCGAGTGTCTGGGATGGGGTTTCCGTCCCGGGACTCCCCTACGAGGGCGGTCCCCGGTA
GCCAGAAGATCCGGCCGGACTCCGAGCCTGGCCCCTTGGGCGCCGTGTAATTCTATAGCT
GTAGGGTTTCCTCTCCAGGGCTGGGTTCCTTCCACTGTAAATCTGGGGTCTTGGATTAGG
TGACCTGCAGCTGCCCTTTGCCCGACTCCAGCAGCTCCTCCGACTCGGCCTCCCCGAGAG
CAGCCCTTCCCGAGTGTCCCTGATCCTGCTCGTCGGCCTCCGCCCAGGCCCCTGTAATCC
GCGCGCCCTCTCCCCGCAGCTGAGGAGAAGTCGTGCGCCGCGGACAGCGACCCGGAGCCT
GAGCGGTTGAGCGAGGAGCGTGCGGGGGCGCCGCTAGGCCGCAGCCCGGCTCCAGACAGC
GCCAGCCCCACTCGCTTGACCGAACCCGAGCGCGCCCGGGAGCGGCGTAGTCCCGAGAGG
GGCAAGGAGCCGGCCGAGAGCGGCGGGGACGGCCCGTTCGGCCTGAGGAGCCTGGAGAAG
GAGCGCGCCGAAGCTCGGAGGAAGGACGAGGGGCGCAAGGAGGCGGCCGAGGGCAAGGAG
CAGGGCCTGGCGCCGCTGGTGGTGCAGACAGACAGTGCGTCCCCCCTGGGCGCCGGACAC
CTGCCCGGCCTGGCCTTTTCCAGCCACTTGCACGGGCAGCAGTTCTTTGGGCCGCTGGGA
GCCGGCCAGCCGCTCTTCCTGCACCCTGGACAGTTCACCATGGGCCCTGGCGCCTTCTCC
GCCATGGGCATGGGTCACCTACTGGCCTCGGTGGCAGGCGGCGGCAACGGCGGAGGTGGC
GGGCCTGGGACCGCCGCGGGGCTGGACGCAGGCGGGCTGGGTCCCGCGGCCAGCGCAGCA
AGCACCGCCGCGCCCTTCCCGTTCCACCTCTCCCAGCACATGCTGGCATCTCAGGTAAGG
CCTGTGACCCCGCGGCAGCGCCAGCGAGGGAGAAGGCCCAGGGAGCTGGCGGCGAGGCCA
GCGGAGGGCCTGAGGGGTGCCAGGGTCGCTGGGATCCTCTGGCAGGCAAGAACTCCATCC
AGGGGAGGCCCCGGGATGCCCTTTAGGACACCTGGGTTCTGAGAGACGAGGACTGTGTTG
TAAGTCCAGGGGCTGGCCAGGGCGCCGCTTCTGACTCCCGTGTGACCTTTGGCAAGTCCC
TGACCCTCTCTGGGCCTGCTTCCTTCCCTATAGCCCCAGCGAGGAGTGCGGTGCCCATCG
AGACTCTTCTCACCCTCACAGACCAGGGCCACCCCTGGCTTGTGAAGTCTTCTCTCCTGG
CCTCAGGACATTTTTCTTGAGAACAAAGACCCTTGGCTCCCTGTCCATTCTGAAAGGCTA
AGGTGGCCAGACAGACAAAGGGAGAAGGAACATTTGCATTATTTTCTGGACAGTTGACCA
CCAGCCCTCCCAGAGGTCTCTGGCCCCTCCCAGGCCTTCTACGAAGTCCTCTGTCCTGGT
GAAGGACAAGGCTGGTCTTTCCCCTACCTTGAGTTCCCAGCACCCCCAGCCACATGTCAG
GGATGAGAACTCAGCTCTTCAGGCCCCATTGAAATTCCAAGCTCCCAGGGGAGTGTTGAC
GAAAATGTGGCTGGGGCACAGTAAGGAGATCCGATGGGGACAGTGGCCTCTTTGGTTGCT
TGGGTGGAATATTCCCTGTGGAAAGATTTGGAATCTCTTCATTCATTTTCTTCCCTCTGA
GGTTTCTGGCAAATTGAACCCCTGAGATCTTATCTCAAGGGAAGGAAAATAGCAGTTCAG

GTCTGAGAAAGCTCCTTTGCTTCAGCAGCAAATATTTTAAAAGTTGGGTTGGTGGGTGGG
GGGGTGGGGGGTTGGGAGGCAGGCGATTCTGAAATGAGTTTTACACACAGCTCAGTGTTA
GGGTGTGTGGAGTGTTTGAGGTATCACCATCACTCATCTGGGGCTCTGGGCTGGAAGGGA
AGCCTCTGGAGAAAGTTCAGGGCCCAGAGGTTTAATCTGCACCCCTGATTATTTGTGTAT
ATGAATGCCTCCTCCCCAGACAAACTGGTAATAATCACCAGCATCACAGAGGTCATGAGG
AAGTCAGGGCTTCTAGACACAGCCTGGCCAACAGGTGCATGACCCGGGTGAGAATACCAG
CACCTGGGAGGCATCCGTTGGTAGGGCTGCTGGTGGTTACATTCCCAGGCCAGTGCAGCC
CATCCCAGACATCAGGTAGAGCCAAATCCCAAGAGAACTGAAGCCTCCCTCCTTCCTCCC
AACCTGGGATGCCAGGTCTGAAATGCCCAGTAGGACCTGACTGTCTCAGGAGGGGGTATT
TTCTCAGGCCTGATGAAGCTGCCTCTTTTCTTCTTCCTGCTAGAGCCAGGCCTGAGGCTC
CCTGGGAGCCCAGTGCAATCATCAGCCCCCTGCCCTCCTCCCCATACCCACTAGCTCTGG
GGAGTAAGCCATTATCTCAAAGGTCAGGCCGTGCACCAGCCAGACCTCATGAACTCAGGA
AGGTGCTTGTCCAGGAGTTCCTGGCTGCTGTGCCCTTCACAGGCAAAGACTGCATTCCTT
CCTCAGCTGCCAGTGAGGTGCTGCCAGGCATTCCCTGTAGAACTTTCAGGCCAGTTTATG
AACTGGTTGGCACCCGTGTCCTCCTCCTGGCCCAGGCAGGAGAACCATGAGCAGGCAGAA
GGAGACTTTGCAAAGTGCCTTCCCCAGCATGTGTGCCCTCTGCCCTTCAGAGCCTGCAGA
TAGGAGGGGTGGCGAGGACACTGTTCTCAATGAGCAGAACCTCCAAGACACCCAAAGCTG
CCTGTTTGCCACCTGGCCCTATGCCTGCCCCGTTTTCTCCCTCAAGGCCTTCACCCGTGC
TAGGGCAGTCACCTGGAATGTCCTTTCCATTACCCCTGCTGTAATGCCCAGCACAGAACT
TGATGGCAGGCCTTTGCATGGTAGCCTGAAGCGATCTCACCCTTCTAACTGGGTTTGGCC
ACAGGCACACTGGCTCATGCTTACCTGTGCTGCCTGTGGTTATAGTTATGCGAATTGTGG
TTTTACATCCCTAAAACAGAAGGGCACGGTGTCCAGGGGATAGCACCCAGCCCAACTTCA
GAAAGACTTCAGGCAAGATGTCTAACCCTCGTCTTGTTCTGTTTCTTCCAGGGAATTCCA
ATGCCCACTTTCGGAGGCCTCTTCCCCTACCCCTACACCTACATGGCAGCAGCAGCCGCA
GCCGCCTCGGCTTTGCCCGCCACTAGTGCTGCAGCTGCCGCCGCCGCAGCCGCCGGCTCC
CTCTCCCGGAGCCCCTTCCTGGGCAGTGCCCGGCCCCGACTGCGTTTCAGCCCCTATCAG
ATCCCGGTCACCATCCCGCCTAGCACTAGCCTCCTCACCACCGGGCTGGCCTCTGAGGGC
TCCAAGGCCGCTGGTGGAAACAGCCGGGAGCCTAGCCCCCTGCCCGAGCTGGCTCTCCGC
AAAGTAGGGGCCCCATCCCGCGGTGCCCTGTCGCCCAGTGGCTCGGCCAAGGAGGCGGCC
AATGAACTGCAGAGCATCCAGAGACTGGTGAGTGGGCTGGAGAGCCAGCGAGCCCTCTCC
CCAGGCCGGGAGTCGCCCAAGTGAGGGGCTGCCCAGCTGCTCCCCTGCCACGCAGGCCAC
CCGGGCTGCCTGCCCCTGCTGCTTGGGACGTGTACAGCACAGAATGAGTATTTATTTAAA
TAAAGGAGAAAAGTGGGCTGCAGCAGCCGGAATAGAGCCTCGTCTGGCAAGTCGGGGCCT
GGGACACTTCCCTGGGCCTCAACAAGGATCAGGCTGCTGGAAACACAGTCACTTGGGAGC
TGCTGGGCTAGGTCCAGATCCGCTCCAGCGTCAAGGTGGCATCCGAAGGTGTCTCTGGTC
TTCCAGCGAGGTGGGAGAGGCCTCATCCAGGGCCCAGCGGTCCCTGCAGAAGCCAGAAGG
TGCAGGGGCCAGGGGTGGGAGCATCGGAGGGAGTCCCAGAGCCCTGGACCTTGGGCCTAG
ACCGCGTGATAAAACTGGGTTGAGGGATGCTGGAACCAGTTACGACTGAAGTCAGTGTAG
ACCTGAGCTGGGAGGGAACCTGTTAGTCTCCCCACCTCTTCCCTGAAGAGACAGGCACCC
CTCCCAGCCGTGGTCAACGGAGGGAGTGGCACTTCTGCCTTGAGTCCCCAGGGGAAAAAA
AAAAAAGATATTTATGAAATAAATGGTAATTTGTGTAAATAAGCTTTAAGGTTCCCAGAA
TATGCAAATTGGTATTAATTTATTCAAAGGTGTACATTGCTGTGTACATATATTTAGAGA
TTAACTCATACATTTAAAGTTTTTTTCATTTTACGTGAGCATCTATATTGTACAGGGCTG
GGGGGGCCCTTGGCTGCGGGAGAAGGCCCAGAGCCCTGGAGGAGCCACCACCCCGCCGGC
CCCTCGACCCCTCGGCCCCTCGGCCCCTCCGCCCGGGTTTGGCTCGCCCGGCCCGCGGGC
TCCACCTCAGGTTTTCACTTTTCGCTCCGGAGCGAGAACGAAACGACAAAAACGCAAGAA
AACAATAAAACGCTAGAAAGCGAAGTGAACGGCGCTGTGCGCTCTGTGGGTCGCGCGGGG
CTGGGGCGCTGCACGCGCGCAAGGAGGGACGAGAGGCGGGGACAAAAGGCTGCGCGCCCC
TCGCCCGGCGGCAGCTGCACCCCGGGGACGGCAGGGCGGCACGGCGGCAGGGCGGCTGGG
GCGCTGGCGGAGCCGCGAGGCCGGGCGGCGCCGGCGCGGGACGCGGGGTGCGTCCCAGGC
CGGGGCTGGACTCCCCCTGCCGGCCGCTCCCGCAGCTCCGCGCAACGGGCCTGGGCACCC
ACCCGGGCCGGGAGGAGAGCAGCCGAGCGGCCTGGACTCCTGGCTTCTGACTCCCTTTCC
GGGAAACCACACTGGAAACCCATCTCCTTTCAATGCTTCTGGGTGGGTGGGCTGGGAGGA
TGGGTGTTCCTTGTCCAACCTCTCCCTTCGGAGGAGGGGTTTGGCTGTGTCCCACCCCCG
TACTCCCCGCCAAATATCGCTGAGCCTAGAATTCTTTCCCCTCTCTCTGCCTCCTGAACC
TCAAAGACAAACATTTCCTCCGCGTCAGGGCTGTTGGCTAGAGTGGCAAAATCTGGACTA
AAAGGTCTTACTTTAACTCATCTA
